# A Stability-Indicating HPLC-DAD Method for Determination of Ferulic Acid into Microparticles: Development, Validation, Forced Degradation, and Encapsulation Efficiency

**DOI:** 10.1155/2015/286812

**Published:** 2015-05-13

**Authors:** Jessica Mendes Nadal, Maria da Graça Toledo, Yasmine Mendes Pupo, Josiane Padilha de Paula, Paulo Vitor Farago, Sandra Maria Warumby Zanin

**Affiliations:** ^1^Postgraduate Program in Pharmaceutical Sciences, Department of Pharmacy, Federal University of Paraná, 632 Lothário Meissner Avenue, 80210-170 Curitiba, PR, Brazil; ^2^Postgraduate Program in Dentistry, Department of Dentistry, State University of Ponta Grossa, 4748 Carlos Cavalcanti Avenue, 84030-900 Ponta Grossa, PR, Brazil; ^3^Postgraduate Program in Pharmaceutical Sciences, Department of Pharmaceutical Sciences, State University of Ponta Grossa, 4748 Carlos Cavalcanti Avenue, 84030-900 Ponta Grossa, PR, Brazil

## Abstract

A simple stability-indicating HPLC-DAD method was validated for the determination of ferulic acid (FA) in polymeric microparticles. Chromatographic conditions consisted of a RP C_18_ column (250 mm × 4.60 mm, 5 *μ*m, 110 Å) using a mixture of methanol and water pH 3.0 (48 : 52 v/v) as mobile phase at a flow rate of 1.0 mL/min with UV detection at 320 nm. The developed method was validated as per ICH guidelines with respect to specificity, linearity, limit of quantification, limit of detection, accuracy, precision, and robustness provided suitable results regarding all parameters investigated. The calibration curve was linear in the concentration range of 10.0–70.0 *μ*g/mL with a correlation coefficient >0.999. Precision (intraday and interday) was demonstrated by a relative standard deviation lower than 2.0%. Accuracy was assessed by the recovery test of FA from polymeric microparticles (99.02% to 100.73%). Specificity showed no interference from the components of polymeric microparticles or from the degradation products derived from acidic, basic, and photolytic conditions. In conclusion, the method is suitable to be applied to assay FA as bulk drug and into polymeric microparticles and can be used for studying its stability and degradation kinetics.

## 1. Introduction

**Figure 1:**
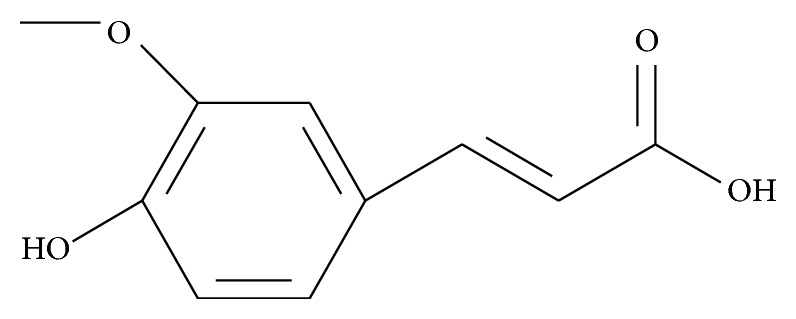
Chemical structure of ferulic acid (FA).

Ferulic acid (FA; C_10_H_10_O_4_; MW: 194.18 Da) or (*E*)-3-(4-hydroxy-3-methoxy-phenyl)prop-2-enoic acid (Figure 1) is a chemical compound widely found in vegetables which has a low degree of toxicity after oral administration [1]. In nature, it is biosynthesized from phenylalanine and l-tyrosine by shikimate pathway and occurs mainly as* trans* isomer [2, 3]. FA is characterized as an off-white to light yellowish brown crystalline powder that has aqueous solubility of 6.63 mg/dL at pH 7.2 [4], pKa of 4.58, melting range between 168 and 171°C, half-life of 42 min [5], and log *P* of 1.67 [6]. In particular, FA has been proposed as a novel antioxidant drug endowed with a strong cytoprotective activity due to both the ability to scavenge free radicals and activate cell stress response [1]. Previous studies have demonstrated that it has diverse therapeutic activity, including anti-inflammatory, antioxidant, antithrombotic, anticancer, cardioprotective, and neuroprotective effects. When used on the skin, FA shows a photoprotective effect [2, 7–11].

In spite of the remarkable pharmacological potential, FA has a poor solubility in aqueous medium [7] and presents an unfavorable pharmacokinetics which reduces its bioavailability and clinical efficacy after oral administration [12]. In order to increase FA bioavailability and enhance its cytoprotective effects, new formulations have been prepared in which this phenolic acid is entrapped either in solid lipid nanoparticles or in niosomes or is bound to other therapeutic agents through organic moieties (e.g., amino acids) which serve as carriers [1]. However, no paper was devoted to investigate the use of polymeric microparticles as a pharmaceutical approach for improving the therapeutic efficacy of FA by prolonging its retention time into the gastrointestinal tract.

Polymeric microparticles are widely studied carriers for the controlled release application of drugs. These multiple-unit pharmaceutical dosage forms are solid and show spherically shaped particles ranging in size from 1 to 1000 *μ*m, in which the drug can be either adsorbed at the surface of the polymer or encapsulated within the polymer [13]. The advantages of using such carriers include potential to increase the bioavailability of poorly water-soluble drugs, ability to control the rate and/or the site of drug release, possibility to improve drug stability related to enzymatic, environmental, or chemical/photochemical degradation, elimination of incompatibilities among different drugs, and as a taste-masking approach [14, 15]. Various methods are readily available for microencapsulation of drugs and one of the most commonly used is emulsion/solvent evaporation [16]. This procedure can be performed via various protocols and the selection for best option depends on the property of the compounds that are intended to be encapsulated [17]. In our laboratory, experiments are being carried out in order to formulate FA-loaded polymeric microparticles using the simple emulsion/solvent evaporation technique and two biocompatible and biodegradable polyesters, poly(*ε*-caprolactone) (PCL) and poly(3-hidroxybutirate-*co*-3-hidroxyvalerate) (PHBV). However, an analytical validation procedure is required for assaying FA from these formulations at the same time.

The literature reports few HPLC methods for determining FA from different sample types as summarized in Table 1. These papers are mainly devoted to describing HPLC methods for isolation and analytical and bioanalytical quantification of FA from medicinal herbs and foods. Only two papers used HPLC method for quantifying FA from inclusion complexes that were prepared using cyclodextrins [2, 18]. However, none of these two studies involving cyclodextrins were submitted to the required validation process. In that sense, no work was found on validation of analytical methods for determining FA as bulk drug and from pharmaceutical formulations.

According to The International Conference on Harmonization (ICH) stability guideline [19], stress testing of a drug substance can be carried out to elucidate its inherent stability characteristics under hydrolytic, oxidative, and photolytic conditions. Considering FA, to the best of our knowledge, there is no stability-indicating method previously published for this drug.

Taking all the above into account, in the current paper, a fast, simple, and optimized reverse-phase HPLC method with UV detection was developed and validated for quantifying FA in polymeric microparticles. Other experiments were also performed to explore forced degradation of the drug under stress conditions as per the ICH guidelines [19–21].

## 2. Materials and Methods

### 2.1. Reagents and Chemicals

Ferulic acid 99.80% (FA, Suzhou Leader Chemical Co. Ltda., Suzhou, China), poly(*ε*-caprolactone) (PCL) ($\overset{-}{M_{w}}$ = 70000–90000 g/mol, Sigma-Aldrich, St. Louis, MO, USA), poly(3-hidroxybutirate-*co*-3-hidroxyvalerate) (PHBV) ($\overset{-}{M_{w}}$ = 380000 g/mol, 8.70 mol% hydroxyvalerate, Biocycle L110, PHB Industrial, Serrana, Brazil), and poly(vinyl alcohol) (PVA) ($\overset{-}{M_{w}}$ = 72000 g/mol, 88.5 mol% of hydrolysis, Vetec, Rio de Janeiro, Brazil) were used as received. As an internal standard,* trans*-ferulic acid, matrix substance ≥99.0% (HPLC), was purchased from Sigma-Aldrich (St. Louis, MO, USA). HPLC-grade methanol was also provided by Sigma-Aldrich (St. Louis, MO, USA). Water was purified in a Milli-Q Plus water purification system (Millipore, Bedford, MA, USA). All other reagents and solvents were of analytical grade.

### 2.2. Equipment

A Varian Pro-star SYS-LC-240-E HPLC system (Walnut Creek, CA, USA) was used for method development. The HPLC system was equipped with a column oven compartment (model ProStar 410), an on-line degasser (model ProStar 230), a ternary pump (model ProStar 230), a solvent delivery module (model ProStar 230), an auto sampler (model ProStar 410), and a photodiode array detector (DAD) (model ProStar 335). Data acquisition, analysis, and reporting were performed using Varian Star Workstation chromatography software (Walnut Creek, CA, USA).

### 2.3. Chromatographic Conditions

Experiments were performed in the previously described HPLC system using a Varian C_18_ analytical column (Walnut Creek, CA, USA) with 5 *μ*m particle size, 4.6 mm internal diameter, and 250 mm length at 25 ± 2°C. The mobile phase consisted of methanol and water adjusted to pH 3.0 with* ortho*phosphoric acid 0.1 N (48 : 52 v/v) at an isocratic flow rate of 1.0 mL/min. The sample injection volume was 10 *μ*L. FA was monitored at 320 nm. The method run time was 8 minutes and all experiments were carried out in triplicate.

### 2.4. Preparation of Polymeric Microparticles

The polyester microparticles containing FA were prepared by simple emulsion/solvent evaporation [22]. Three different formulations (Table 2) were obtained for each polymer (PHBV/PCL) depending on the amount of FA into their compositions (5, 10, and 20%). Chloroform and methylene chloride were used as polymer solvent for PHBV and PCL, respectively. Briefly, the organic phase was added into the aqueous phase under mechanical stirring (5000 rev/min) for 5 min. The emulsion was kept under mechanical stirring (800 rev/min) at room temperature (25 ± 2°C) for 4 h. After organic solvent evaporation, microparticles were separated by centrifugation (2500 rev/min, 10 min), washed twice with purified water, and dried under vacuum at 35 ± 2°C for 4 h. The samples were stored into a desiccator under vacuum at room temperature (25 ± 2°C). All formulations were obtained in triplicate. Unloaded-microparticles were also prepared as negative controls (M1FA0 and M2FA0). All procedures were performed in dark conditions.

### 2.5. Preparation of Standard Solutions

A stock standard solution (1 mg/mL) was daily prepared by dissolving 50 mg of FA into a 50 mL volumetric flask using methanol. This solution was further diluted in methanol to prepare seven different working standard solutions ranging from 10.0 to 70.0 *μ*g/mL. These solutions were filtered through a poly(vinylidene fluoride) membrane filter (Durapore membrane, 0.45 *μ*m pore size, Millipore, Bedford, MA, USA) before injection into the HPLC system. All procedures were carried out in dark conditions.

### 2.6. Preparation of Sample Solutions

For FA quantification, the amount of drug into FA-loaded PHBV/PCL microparticles was indirectly determined. As previously described, microparticles were centrifuged at 2500 rev/min for 10 minutes and washed twice with purified water after organic solvent evaporation. Free FA was assayed in the supernatant after its suitable dilution and filtration through a 0.45 *μ*m poly(vinylidene fluoride) membrane filter by injection into the HPLC system.

### 2.7. Method Development

Detection wavelength for the HPLC study was selected as 320 nm. The chromatographic conditions were optimized for resolution of the peak of FA by varying the composition and proportion of the mobile phase. Samples of different formulations were used to optimize the chromatographic conditions for resolving FA. An appropriate blank was injected before the analysis of all samples. The method was then validated and used for the determination of FA into PHBV/PCL microparticles.

### 2.8. Method Validation

Validation studies were performed using the optimized chromatographic conditions based on the principles of validation described in the International Conference Harmonization (ICH) guidelines [21]. The method was validated for specificity, linearity, limit of detection (LOD), limit of quantitation (LOQ), accuracy, precision, and robustness.

The specificity was determined by analyzing the chromatograms of unloaded microparticles (M1FA0 and M2FA0) in comparison with those obtained for FA-loaded microparticles (M1FA10 and M2FA10) aiming at confirming that none of the excipients interfere with the quantitation of the drug.

The linearity was determined by calculating a regression line from the plot of the peak area* versus* concentration of the working standard solutions prepared at seven concentration levels (10.0, 20.0, 30.0, 40.0, 50.0, 60.0, and 70.0 *μ*g/mL) using least-squares linear regression analysis. The linearity test was performed for 3 consecutive days in the same concentration range. The solutions were injected in triplicate into the HPLC column keeping the injection volume constant (10 *μ*L) and chromatograms were recorded. The standard deviation (SD) value for the slope and *Y*-intercept of the calibration curve were calculated.

LOD and LOQ were calculated based on the standard deviation of the response (*δ*) and the slope (*S*) of the calibration curve and were expressed as 3.3 *δ*/*S* and 10 *δ*/*S* for LOD and LOQ, respectively.

The accuracy of the analytical method was investigated by spiking unloaded microparticles (M1FA0 and M2FA0) with known concentrations of the stock solution to achieve final theoretical drug concentrations of 15, 45, and 65 *μ*g/mL. The accuracy value was determined by calculating the percent recovery of FA for these three concentration levels and then determining the relative standard deviation (RSD).

The precision was assessed at two levels: repeatability (intraday precision) and intermediate precision (interday precision) using M1FA10. The repeatability was investigated by testing three different sample solutions at 15, 40, and 65 *μ*g/mL on the same day. Three samples solutions at 40 *μ*g/mL were also evaluated in other two different days in order to determine intermediate precision. Results were reported in terms of RSD.

In order to determine the robustness, experimental conditions were purposely changed to check the reproducibility of the method. Robustness was evaluated by analyzing drug content of the microparticles (M1FA10) with variations in the temperature of analytical column (30 and 40°C), flow rate (0.9 and 1.1 mL/min), and pH of mobile phase (4.0 and 6.0). Samples were evaluated in triplicate for each variation of the method conditions. Chromatograms were recorded and compared with the previously reported chromatographic conditions.

### 2.9. Forced Degradation Study

Forced degradation studies were also carried out in order to provide some information about drug stability and specificity of the proposed method. The standard solution of FA was subject to accelerated degradation by acid, basic, and photolytic conditions.

To investigate the acid degradation, 6.25 mL of the stock standard solution (1.0 mg/mL) was diluted into a 25 mL volumetric flask with 0.1 M HCl. This solution was maintained at room temperature (25 ± 2°C) and protected from light for 1 h. After the reaction time, the solution was neutralized using 0.1 M NaOH. The solution was diluted with methanol to achieve a final concentration of 40 *μ*g/mL before injection into the HPLC system. The same procedure was used for the alkaline degradation, except by the fact that 0.1 M HCl was replaced by 0.1 M NaOH in sample preparation.

For photodegradation, 3 mL of FA methanol solution (1 mg/mL) was placed in a quartz cuvette and subsequently exposed to UV radiation (Phillips TUV lamp–254 nm, 30 W) for 6 h in a mirrored chamber (1 m × 25 cm × 25 cm) at a fixed distance. At predetermined times (0, 1, 2, 3, 4, 5, and 6 h) of exposure to light, 400 *μ*L of the samples were withdrawn and diluted with methanol (final concentration of 40 *μ*g/mL of FA) in order to quantify the remaining FA according to the method previously described. In order to refute the hypothesis of thermal degradation, a cuvette containing FA methanol solution (final concentration of 40 *μ*g/mL of FA) was covered by aluminum paper and was evaluated as the same way. The degradation rate kinetics of FA was determined and the best fit was used for indicating the reaction order. The kinetic models used were zero order (*C* = *C*_o_ − kt), first order (ln* C* = ln *C*_o_ − kt), and second order equation (1/*C* = 1/*C*_o_ + kt).

### 2.10. Evaluation of Encapsulation Efficiency

In order to demonstrate the applicability of the validated method, the encapsulation efficiency (EE) of FA into PHBV/PCL microparticles was calculated using (1) from the HPLC results provided by sample solutions:

(1) EE%=total  drug  content−free  drug  contenttotal  drug  content×100.

## 3. Results and Discussion

### 3.1. Method Development and Optimization

Previous tests were carried out in order to provide a quick and effective method for analyzing FA using HPLC. The investigated chromatographic conditions were mainly related to the mobile phase composition. Initial runs were performed using a mobile phase mixture of acetonitrile : water (16 : 84 v/v) containing 1% glacial acetic acid based on a previously reported method for FA quantification in plasma [23]. However, irregular peaks were observed, showing low chromatographic resolution probably due to instrumental and column differences.

In order to improve the quality of chromatographic method, various ratios in isocratic mode were tested using different mixtures of acetonitrile : methanol : water adjusted to pH 3.0 with acetic acid. These assays demonstrated that the proportion of acetonitrile : methanol : water adjusted to pH 3.0 with acetic acid (24 : 24 : 52 v/v) was more appropriate for the method optimization. In spite of the symmetry of FA peak, no repeatability and accuracy were observed using this mobile phase.

**Figure 2:**
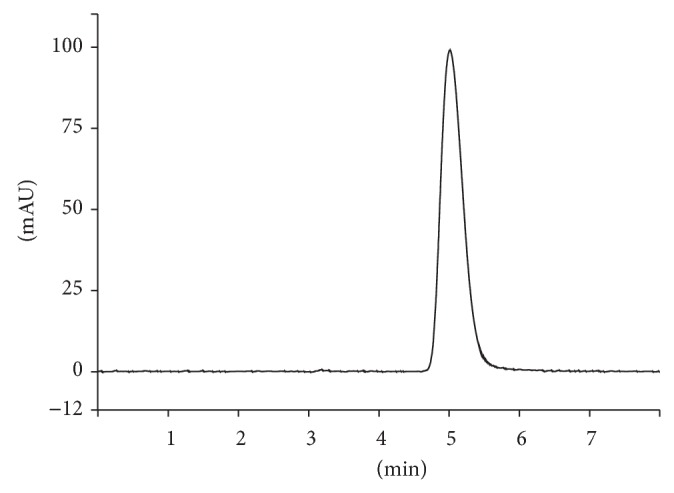
Representative HPLC chromatogram of ferulic acid standard (40 *μ*g/mL) in methanol : water adjusted to pH 3.0. Mobile phase: methanol : water adjusted to pH 3.0 using* ortho*phosphoric acid (48 : 52 v/v); flow rate: 1.0 mL/min; detection wavelength: 320 nm; column temperature: 25 ± 2°C; and injection volume: 10 *μ*L.

Therefore, additional experiments were carried out removing acetonitrile and using acetic acid and* ortho*phosphoric acid as acidifying agents at different pH values. The mobile phase composed by methanol : water adjusted to pH 3.0 using* ortho*phosphoric acid (48 : 52 v/v) provided a lower tailing and a more symmetric peak for FA with a lower retention time (theoretical plates = 1253, *k*′ = 3.77, and tail factor = 1.3). The peak was detected at 4.86 min (Figure 2) which is very suitable for routine analyses.

In summary, methanol : water adjusted to pH 3.0 using* ortho*phosphoric acid (48 : 52 v/v), column temperature at 25 ± 2°C, sample injection volume of 10 *μ*L, isocratic flow rate of 1.0 mL/min, detector set at 320 nm, and run time of 8 minutes were chosen as suitable chromatographic conditions for further procedures including the method validation.

### 3.2. Method Validation

The proposed method was validated by determining its performance characteristics regarding specificity, linearity, limit of detection, limit of quantification, accuracy, precision, and robustness [21].

#### 3.2.1. Specificity

**Figure 3:**
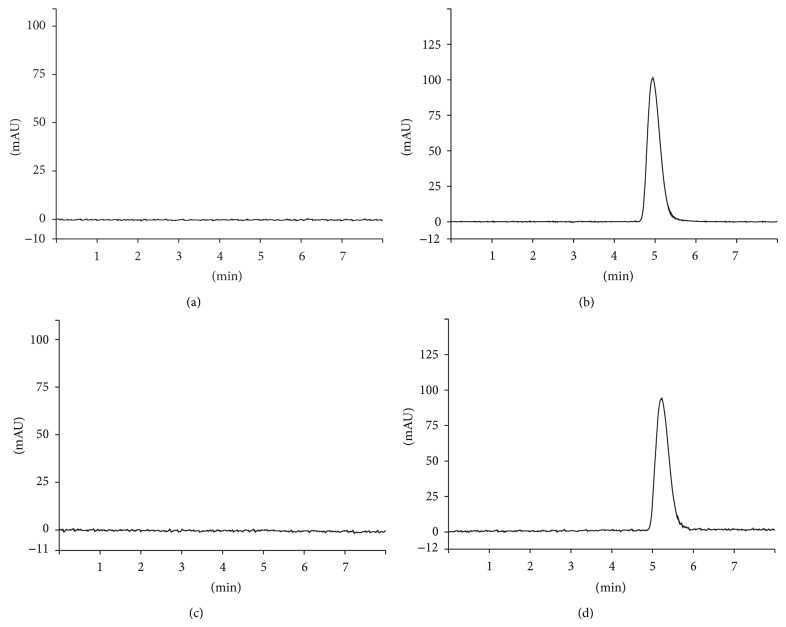
HPLC chromatograms (*λ* = 320 nm) obtained from unloaded and FA-loaded microparticles: M1FA0 (a), M1FA10 (b), M2FA0 (c), and M2FA10 (d).

Specificity was demonstrated by comparing the chromatograms of unloaded and FA-loaded microparticles prepared as per test method. The results showed that there was no interference at the retention time of FA from the other formulation components. In that sense, it is possible to confirm the specificity of the purposed method (Figure 3). Moreover, the photodiode array detector indicated that FA peak was free from interference (purity index > 0.9999).

#### 3.2.2. Linearity

**Figure 4:**
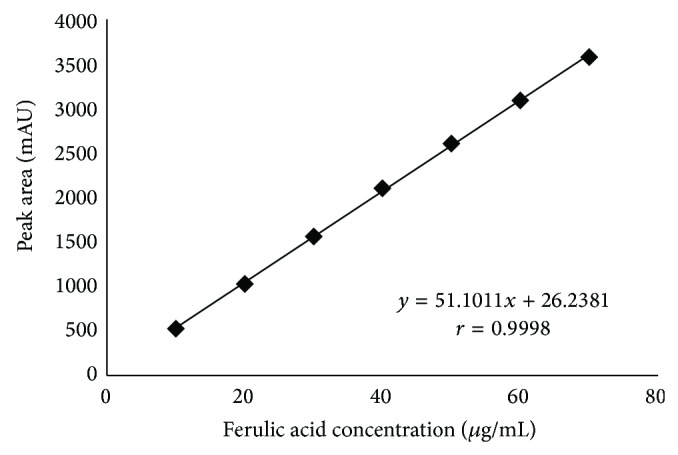
Mean calibration curve obtained for FA using working standard solutions at the concentration range of 10.0 to 70.0 *μ*g/mL (*n* = 3).

A linear relationship between peak area and concentration of FA at the concentration range of 10.0 to 70.0 *μ*g/mL was observed (Figure 4). The linear equation obtained by the least-square method was *y* = 51.1011*x* + 26.2381, where *y* is the peak area and *x* is the standard solution concentration in *μ*g/mL. A suitable correlation coefficient (*r* = 0.9998) was recorded which demonstrates that the method is remarkable linear with an *r* value of nearly 1 at the purposed range.

#### 3.2.3. Limit of Detection (LOD) and Limit of Quantification (LOQ)

The lowest concentration where FA can be detected (LOD) and quantified (LOQ) with acceptable precision and accuracy was 0.334 and 1.012 *μ*g/mL, respectively. These results represent that the chromatographic method is suitable enough to detect and quantify FA at the concentration range of 10.0 to 70.0 *μ*g/mL.

#### 3.2.4. Accuracy

The accuracy was evaluated using a recovery study and showed mean recoveries for the three levels of concentration ranging between 99 and 101% (Table 3). These recovery values indicate that the developed method was accurate for the determination of FA in polymeric microparticles.

#### 3.2.5. Precision

Precision was verified by repeatability and intermediate precision as presented in Table 4. The relative standard deviation (RSD) values were less than 1.43% and 1.93% for intra- and interday precision, respectively. These results confirm the good precision of the chromatographic method.

#### 3.2.6. Robustness

The evaluation of robustness was based on RSD values obtained by changing analytical parameters such as temperature of analytical column (30 and 40°C), isocratic flow rate (0.9 and 1.1 mL/min), and pH of mobile phase (4.0 and 6.0). Concerning these parameters, the method was considered robust because RSD for the drug content analyses values were lower than 2.24% as summarized in Table 5. Therefore, changes in these chromatographic parameters did not affect the analysis of FA into polymeric microparticles. As expected, some variation in the retention time was observed without compromising the determination of drug content.

### 3.3. Forced Degradation Study

**Figure 5:**
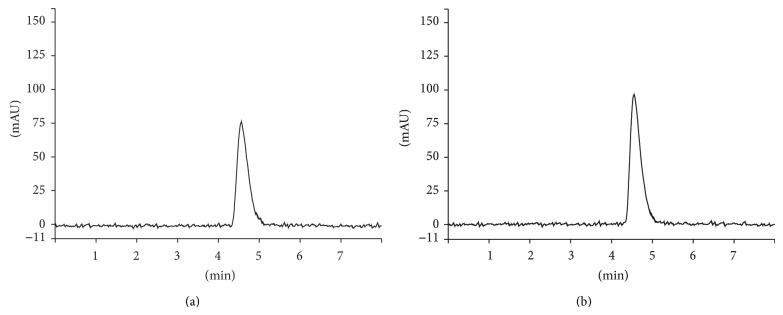
Chromatograms obtained after one hour of FA exposure under acid (a) and alkaline (b) conditions.

**Figure 6:**
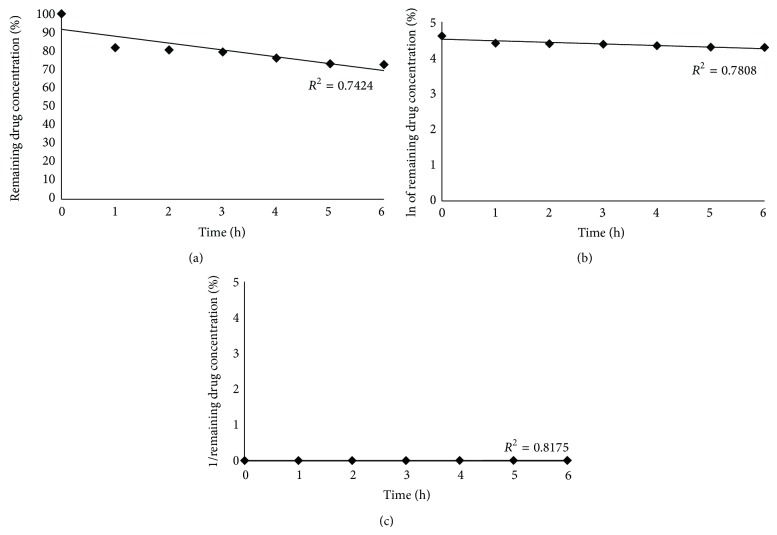
Plots of remaining FA concentration* versus* time for zero order (a), first order (b), and second order (c) equations.

Regarding the presence of degradation products, forced degradation study showed different results depending on the stress condition used. After exposure to acid medium, approximately 29.37% of FA was degraded (Figure 5(a)) and the drug peak was recorded at 4.56 min. In alkaline condition, a FA degradation of 16.33% was observed and the drug peak was detected at 4.55 min (Figure 5(b)). No previous report was devoted to investigate the drug degradation using 0.1 M HCl and 0.1 M NaOH. Under photolytic conditions, decreases in FA concentration from methanol solution were observed (Figure 6). On the other side, the cuvette covered by aluminum paper showed no degradation after 6 h which indicates that no thermal degradation was involved in the photodegradation process. Moreover, no additional peak was verified in the chromatograms of the forced degradation study demonstrating that the degradation products were not detected using the optimized chromatographic conditions.

Aiming at elucidating the kinetics of FA photodegradation from methanol solution, the experimental data were fitted to zero, first, and second order equations. These plots (Figure 6) indicated that FA photodegradation process in methanol solution followed a second order kinetic with a rate constant of 6.27 × 10^4^ L/mol × h. In this case, the degradation of FA is dependent on the drug concentration.

### 3.4. Evaluation of Encapsulation Efficiency

The drug content and encapsulation efficiency (EE) of FA into PHBV/PCL microparticles was carried out by the previously validated HPLC-DAD method and the obtained results are represented in Table 6. High percentages of drug entrapment were obtained for PHBV/PCL microparticles by simple emulsion/solvent evaporation. All formulations showed suitable EE values higher than 98%.

These values are mainly based on the poor aqueous solubility (6.63 mg/dL at pH = 7.2) of FA [4] which leads to increase the drug loaded into polymeric microparticles. The current results are similar or better than previously reported. Stearic acid- and stearyl ferulate-based solid lipid nanoparticles containing* trans*-FA revealed a drug entrapment of 95.4 and 97.7%, respectively [12]. Poly(lactic-*co*-glycolic acid) nanoparticles containing FA showed EE of about 76% [7]. A FA entrapment higher than 60% was achieved for inclusion complex of* trans*-FA and hydroxypropyl-*β*-cyclodextrin that was prepared by the freeze-drying method [2].

Therefore, the validated method was successfully applied to the determination of FA into polymeric microparticles and can be considered an important tool for the quality control of these promising formulations.

## 4. Conclusion

A simple and efficient reverse-phase HPLC-DAD method was developed and validated for quantitative determination of FA into polymeric microparticles. In summary, the method was found to be specific, linear, accurate, precise, and robust for a rapid determination of this drug and can be used for studying the stability and degradation kinetics of FA.

## Figures and Tables

**Table 1:** Previous papers that report HPLC methods for isolation and quantification of FA.

Sample type	Chromatographic conditions	Was the (bio)analytical validation process carried out?	Reference
Rat plasma after oral administration of Chuanxiong rhizome and its related preparation (Suanzaoren)	Column: Hypersil C_18_ column (200 × 4.6 mm, 5 *μ*m); mobile phase: acetonitrile : water (16 : 84 v/v) containing 1% glacial acetic acid; injection volume: 20 *μ*L; flow rate: 0.8 mL/min; column temperature: ambient; detection: UV (320 nm); retention time: 12.7 min.	Yes	[23]

Ferulic acid/*γ*-cyclodextrin inclusion complex	Column: Supelcosil LC-18 (250 × 4.6 mm, 5 *μ*m); mobile phase: acetonitrile : water (2% acetic acid) (22 : 78 v/v); injection volume: 20 *μ*L; flow rate: 1.0 mL/min; column temperature: ambient; detection: UV (320 nm); retention time: 10.5 min.	No	[18]

Rabbit plasma after intravenous administration of danxiongfang (a Chinese medicine formula used to treat atherosclerosis and vascular restenosis)	Column: Agilent HC-C_18_ (250 × 4.6 mm, 5 *μ*m); mobile phase: methanol : water (0.5% glacial acetic acid) (20 : 80 to 80 : 20 v/v); injection volume: 20 *μ*L; flow rate: 1.0 mL/min; column temperature: ambient; detection: UV (281 nm); retention time: 7.78 min.	Yes	[24]

Ma-Zu-Wan-Show-Yao-Jyo (a Chinese tonic wine)	Column: Cosmosil 5C_18_-AR-II (250 × 4.6 mm, 5 *μ*m); mobile phase: water adjusted to pH 3.0 with 0.1 N phosphoric acid : acetonitrile (65 : 35 to 75 : 25 v/v); injection volume: 20 *μ*L; flow rate: 1.0 mL/min; column temperature: ambient; detection: UV (285 nm); retention time: 10.0 min.	Yes	[25]

Xuebijing injection (a traditional Chinese medicine for treating sepsis)	Column: Zorbox SB C_18_ (250 × 4.6 mm, 5 *μ*m); mobile phase: 0.2% phosphoric acid : acetonitrile in gradient elution; injection volume: 20 *μ*L; flow rate: 1.0 mL/min; column temperature: ambient.	Yes	[26]

Rice (*Oryza sativa* L.)	Column: C_18_ Waters Symmetry column (150 × 3.9 mm, 5 *μ*m) coupled to a guard column; mobile phase: 1% (v/v) acetic acid : acetonitrile in gradient elution; injection volume: 20 *μ*L; flow rate: 0.8 mL/min; column temperature: 30°C; detection: UV (280 nm); retention time: 18 min.	Yes	[27]

Ferulic acid/hydroxypropyl-*β*-cyclodextrin inclusion complex	Column: C_18_ Symmetry column (150 × 3.9 mm; 5 *μ*m); mobile phase: methanol : water : acetic acid (50 : 50 : 0.5); injection volume: 10 *μ*L; flow rate: 0.8 mL/min; column temperature: 30°C; detection: UV (313 nm); retention time: 15 min.	No	[2]

Insampaedok-san (a traditional oriental medicine prescription for treating cold-related symptoms)	Column: Shiseido C_18_ column (250 × 4.6 mm, 5 *μ*m); mobile phase: water with 0.1% trifluoroacetic acid : methanol in gradient elution; injection volume: 20 *μ*L; flow rate: 1.0 mL/min; column temperature: 35°C; detection: UV (320 nm); retention time: 22.07 min.	Yes	[28]

Nao-De-Sheng (a tradicional Chinese formula containing Chuanxiong rhizome)	Column: C_18_ column (250 × 4.6 mm, 5 *μ*m); mobile phase: methanol : water (0.5% acetic acid) (30 : 70 v/v); injection volume: 20 *μ*L; flow rate: 1.0 mL/min; column temperature: 35°C; detection: UV (320 nm); retention time: 15.02 min.	Yes	[29]

*Ferula asafoetida* and a polyherbal preparation	Column: HiQSil ODS C-18 (250 × 4.6 mm, 5 *μ*m); mobile phase: acetonitrile: 10% acetic acid (20 : 80 v/v), pH 2.25; injection volume: 20 *μ*L; flow rate: 1.0 mL/min; column temperature: 30°C; detection: UV (319 nm); retention time: 10.24 min.	Yes	[30]

**Table 2:** Composition of ferulic acid-loaded and unloaded PHBV/PCL microparticles.

Composition	Formulation							
	M1FA0	M1FA5	M1FA10	M1FA20	M2FA0	M2FA5	M2FA10	M2FA20
Aqueous phase								
Polysorbate 80 (g)	0.25	0.25	0.25	0.25	0.25	0.25	0.25	0.25
PVA (g)	4.00	4.00	4.00	4.00	4.00	4.00	4.00	4.00
Purified water (mL)	200.0	200.0	200.0	200.0	200.0	200.0	200.0	200.0
Organic phase								
Ferulic acid (g)	—	0.10	0.20	0.40	—	0.10	0.20	0.40
PHBV (system M1) (g)	2.00	1.90	1.80	1.60	—	—	—	—
PCL (system M2) (g)	—	—	—	—	2.00	1.90	1.80	1.60
Chloroform (mL)	40.0	40.0	40.0	40.0	—	—	—	—
Methylene chloride (mL)	—	—	—	—	40.0	40.0	40.0	40.0

**Table 3:** Accuracy assays for ferulic acid analysis^∗^.

Level of concentration	Theoretical concentration	Experimental concentration	Recovery (%)	RSD^∗∗^ (%)
	(*µ*g/mL)	(*µ*g/mL)		
Low	15	15.11 ± 0.25	100.73	1.65
Medium	45	44.91 ± 0.44	99.80	0.98
High	65	64.36 ± 0.55	99.02	0.85

^∗^ *n* = 3; ^∗∗^RSD = relative standard deviation.

**Table 4:** Repeatability and intermediate precision data for ferulic acid analysis.

Sample solution (*μ*g/mL)	Measured concentration ± SD^∗^ (*μ*g/mL)	RSD^∗∗^ (%)
Repeatability (*n* = 9)		
15	16.82 ± 0.24	1.43
40	40.21 ± 0.31	0.77
65	64.67 ± 0.30	0.46
Intermediate precision (*n* = 3)		
Day 2		
40	39.44 ± 0.05	0.13
Day 3		
40	40.32 ± 0.78	1.93

^∗^SD = standard deviation; ^∗∗^RSD = relative standard deviation.

**Table 5:** Robustness data for ferulic acid analysis (*n* = 3).

Parameter	Drug content (%) ± SD^∗^	RSD^∗∗^	Retention time (min)
Flow rate (mL/min)			
0.9	102.71 ± 1.93	1.88	5.39
1.0	100.12 ± 0.60	0.60	4.86
1.1	99.78 ± 0.57	0.57	4.49
pH of mobile phase			
3.0	100.12 ± 0.60	0.60	4.86
4.0	97.56 ± 0.98	1.00	4.80
6.0	98.03 ± 0.37	0.38	4.52
Temperature (°C)			
Ambient	100.12 ± 0.60	0.60	4.86
30	100.09 ± 1.09	1.09	4.71
40	99.14 ± 2.22	2.24	4.44

^∗^SD = standard deviation; ^∗∗^RSD = relative standard deviation.

**Table 6:** Ferulic acid-loaded^∗^ and encapsulation efficiency (EE) for PHBV/PCL microparticles.

Microparticles	Ferulic acid-loaded (mg·g^−1^)	EE (%)
M1FA5	49.51 ± 0.21	99.02
M1FA10	100.12 ± 0.60	100.12
M1FA20	198.65 ± 0.97	99.32
M2FA5	49.35 ± 1.76	98.70
M2FA10	98.70 ± 2.06	98.70
M2FA20	198.20 ± 2.45	99.10

^∗^Mean (*n* = 3) ± standard deviation.
